# Does implant weight matter? The effect of total knee arthroplasty on thigh mass distribution and estimated hip loading

**DOI:** 10.1002/jeo2.70925

**Published:** 2026-09-25

**Authors:** Roberto Rossi, Federica Rosso, Mattia Sisella, Francesco Romano, Tommaso Nosenzo, Bernardo Innocenti

**Affiliations:** ^1^ Department of Orthopaedics and Traumatology, AO Ordine Mauriziano University of Torino Torino Italy; ^2^ Department of Orhtopedics University of Torino Torino Italy; ^3^ BEAMS Department Universitè Libre de Bruxelles Bruxelles Belgium

**Keywords:** centre of mass, hip muscle moment, knee arthroplasty, weight

## Abstract

**Purpose:**

Few studies in the literature have evaluated the effect of weight changes on knee joint biomechanics induced by total knee arthroplasty (TKA). The aim of this study was to evaluate changes in the hip muscular moment and the thigh centre of mass following primary TKA.

**Methods:**

All primary TKAs performed with conventional instrumentation between April and December 2024 were prospectively included. Radiographic measurements were obtained to develop an analytical biomechanical model. The resected bone was collected and weighed intraoperatively, together with the femoral component and implanted cement. An analytical biomechanical model was developed to estimate changes in thigh weight, centre of mass position and hip muscular moment before and after TKA.

**Results:**

A total of 70 patients were included in the study. Primary TKA resulted in a statistically significant increase in thigh weight and a distal shift of the thigh centre of mass (*p* < 0.05). The average increase in implant‐related knee weight after TKA was 333.5 g (standard deviation [SD] 51.0 g), while the femoral weight increased by 199.1 g (SD 40.1 g), resulting in an average increase in total thigh weight of 2.8% (SD 0.6%; range: 1.6–4.2%). The analytical biomechanical model showed a statistically significant distal shift of the thigh centre of mass of 6.4 mm (SD 1.5 mm; range: 3.7–9.7 mm) after TKA (*p* < 0.05). This redistribution of thigh mass was associated with a statistically significant estimated increase in hip muscular moment of 6.3% (SD 1.4%; maximum increase 9.3%; *p* < 0.05).

**Conclusion:**

Primary TKA alters thigh mass distribution through an increase in limb weight and a distal shift of its centre of mass. Within the assumptions of this analytical model, these changes result in an increase in estimated hip muscular moment. Further biomechanical and clinical studies are needed to determine the clinical relevance of these theoretical findings.

**Level of Evidence:**

Level IV.

## Graphical abstract

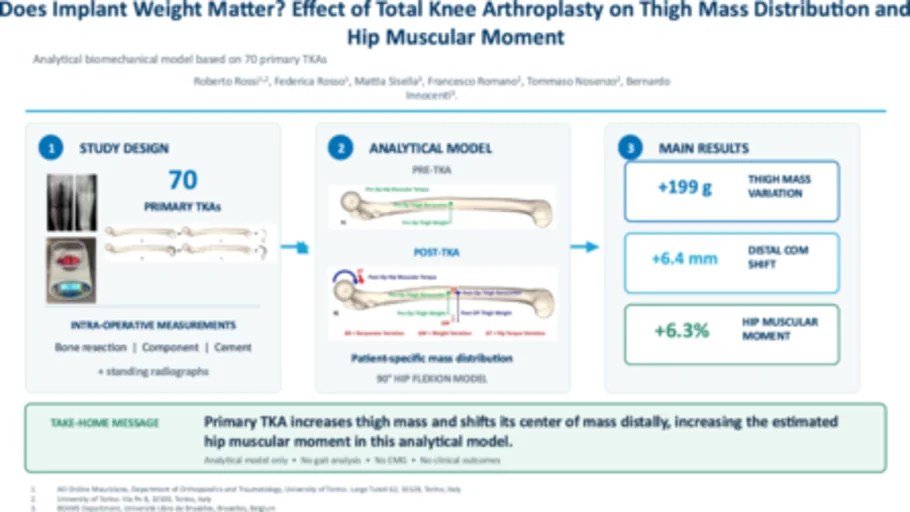

**Abbreviations**
BMI: body mass index
FAL: femoral anatomical length
FML: femoral mechanical length
HKA: hip–knee–ankle angle
MC: medial congruent
PS: posterior‐stabilized
SD: standard deviation
S‐JL: ASIS‐joint line distance
TKA: total knee arthroplasty

## INTRODUCTION

Total knee arthroplasty (TKA) is one of the most frequently performed orthopaedic procedures worldwide [13], with satisfactory long‐term outcomes and survival rates [7, 15, 25].

However, despite these encouraging outcomes, 10%–20% of patients continue to report chronic pain or dissatisfaction after TKA, even in the absence of clinical or radiological signs of failure [2, 5, 8].

There are multiple contributors to persistent symptoms after TKA, possibly including increased patellofemoral forces due to component alignment [21], given that up to 85% of patients localize their pain anteriorly [16]. Several strategies to reduce the number of dissatisfied patients have recently been introduced, including uncemented implants, different alignment philosophies [19] and particular attention to the so‐called ‘third space’. However, patients with unexplained dissatisfaction are still present, and the treatment of this condition can be challenging [17].

One of the potential factors contributing to pain after knee arthroplasty, in the absence of identifiable mechanical causes, may be related to the implant itself and to the increase in femoral weight following TK. Previous studies have demonstrated an increase in lower‐limb weight after TKA implantation. Gibon et al. reported a mean in lower‐limb weight increase after TKA of about 270 g [6], while Zhu et al. reported an average increased weight of about 300 g [29]. Furthermore, Zhu et al. also found a negative correlation between the weight increase after TKA implantation and immediate post‐operative Hospital for Special Surgery score [29]. Although the absolute increase in weight is modest, it remains poorly understood [18]. Some studies have demonstrated that increases in total body mass substantially elevate the muscular forces required to perform basic functional tasks, including walking, stair climbing and sit‐to‐stand transitions [1, 12].

Similarly, a segmental mass variation in the femur after TKA implantation may alter joint moments and lever arms, especially in patients for whom the weight increase is greater, potentially leading to increased muscular workload. Hypothetically, this could lead to anterior knee pain, early fatigability, altered gait strategies or the subjective perception of a ‘heavier’ limb reported by some patients after TKA [4, 11, 23].

However, the biomechanical role of post‐operative thigh mass augmentation—particularly its impact on hip flexor demand—has not yet been clearly defined. The aim of this study was to quantify the increase in thigh weight following TKA and to explore, with an analytical model, its potential biomechanical consequences on hip flexor function during everyday activities. We hypothesized that TKA would result in a non‐negligible increase in thigh weight and that this additional mass would increase the biomechanical demand on the hip flexor muscles.

## MATERIALS AND METHOD

### Study design, patient population and surgical procedure

This study prospectively collected clinical data and combined them with an analytical biomechanical model.

All primary TKAs performed with the same implant using conventional instrumentation from April 2024 to December 2024 were prospectively included.

Inclusion criteria were (1) primary cemented TKA, (2) patients able to provide informed consent, (3) posterior‐stabilized (PS) or medial congruent (MC) inserts. Exclusion criteria were (1) previous surgeries other than simple arthroscopy, (2) previous hardware in the lower limb, (3) severe renal disease or other pathology significantly affecting bone density, (4) patellar replacement, (5) additional long stems (excluded the short 30 mm stem suggested for the MC insert) or augments and (6) revision surgery.

All patients underwent primary cemented TKA. The same implant family (Persona®; Zimmer Biomet) was used in all cases, with either a PS or MC configuration according to the predefined indication, with no patellar replacement. All surgeries were performed by the same surgeon (R.R.) with the aim of achieving mechanical alignment. Surgeries were performed under spinal anaesthesia through a medial parapatellar approach. Soft‐tissue removal was kept to a minimum to avoid influencing post‐operative thigh weight and was limited to synovial tissue required for accurate positioning of the anterior femoral stylus, together with resection of the cruciate ligaments. MC inserts were used in case of neutral or varus alignment or in young high‐demand patients, while PS implants were used in older low‐demand patients or in case of valgus alignment. When a MC insert was used, a 30‐mm short stem was added to enhance tibial baseplate fixation. The cementation technique was the same for all patients, using full cementation at both the implant and bone interfaces.

### Data collection

Patient data were anonymized, except for sex, body mass index (BMI) and knee morphotype. A total of 70 patients were included in this study: 43 females (61.4%), with a mean BMI of 28.2 kg/m^2^ (standard deviation [SD] 4.7).

**Figure 1:**
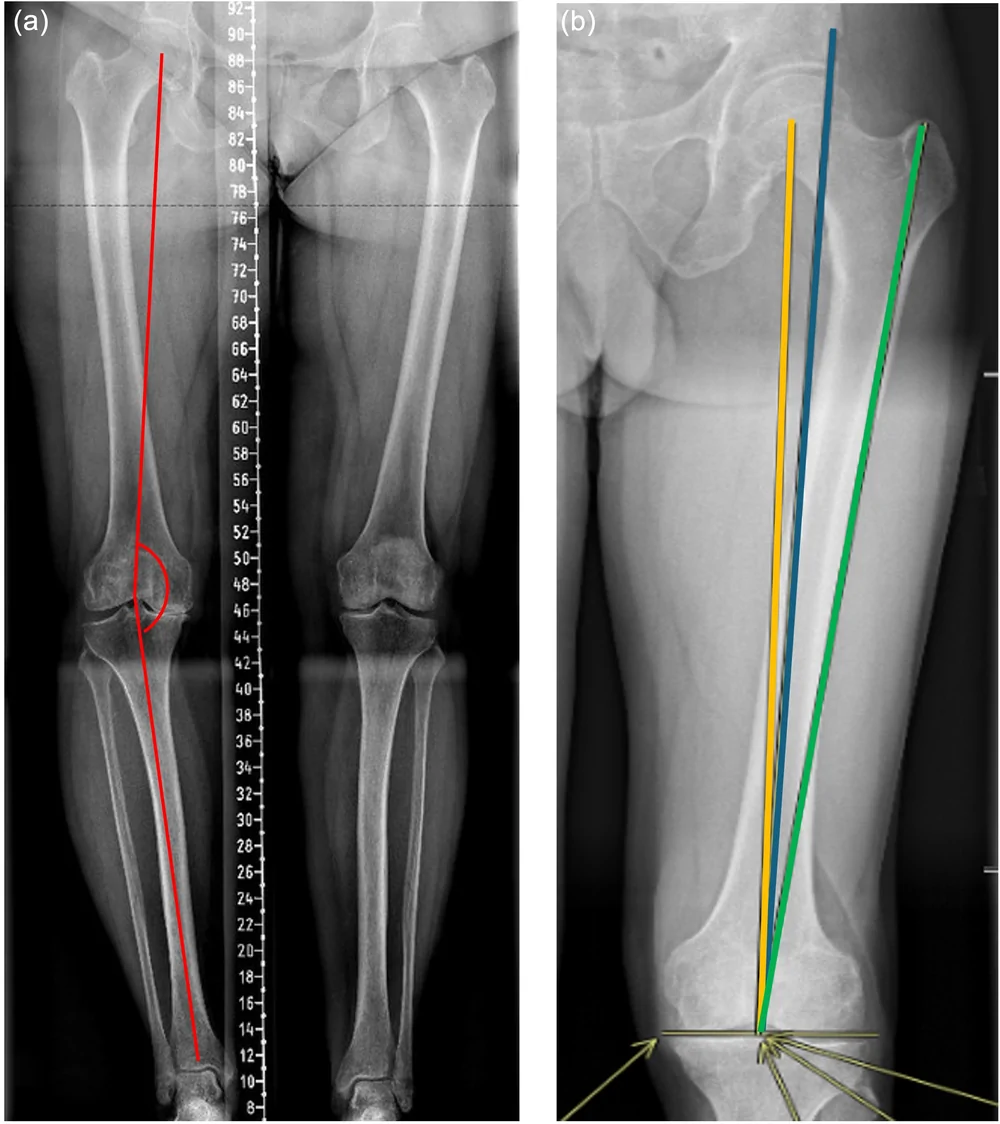
X‐ray demonstrating the different measures performed to obtain the biomechanical model. (a) Hip–knee–ankle (HKA) angle (red line). (b) Yellow line: the femoral mechanical length; blue line: the ASIS‐Joint line distance; green line: the femoral anatomical length. ASIS, anterior superior iliac spine.

Pre‐operative radiographic measurements used for the biomechanical model were obtained from standard long‐leg radiographs by the same observer (Figure 1):

- Hip–knee–ankle angle (HKA): the angle between a line through the centre of the femoral head and the centre of the knee and a line through the centre of the knee and the centre of the ankle
- Femoral mechanical length (FML, mm): the distance between the centre of the femoral head and the centre of the knee joint
- Femoral anatomical length (FAL, mm): the distance between the tip of the greater trochanter and the centre of the knee joint
- ASIS‐joint line distance (S‐JL, mm): distance between the anterior superior iliac spine (ASIS) and the femoral joint line.

**Figure 2:**
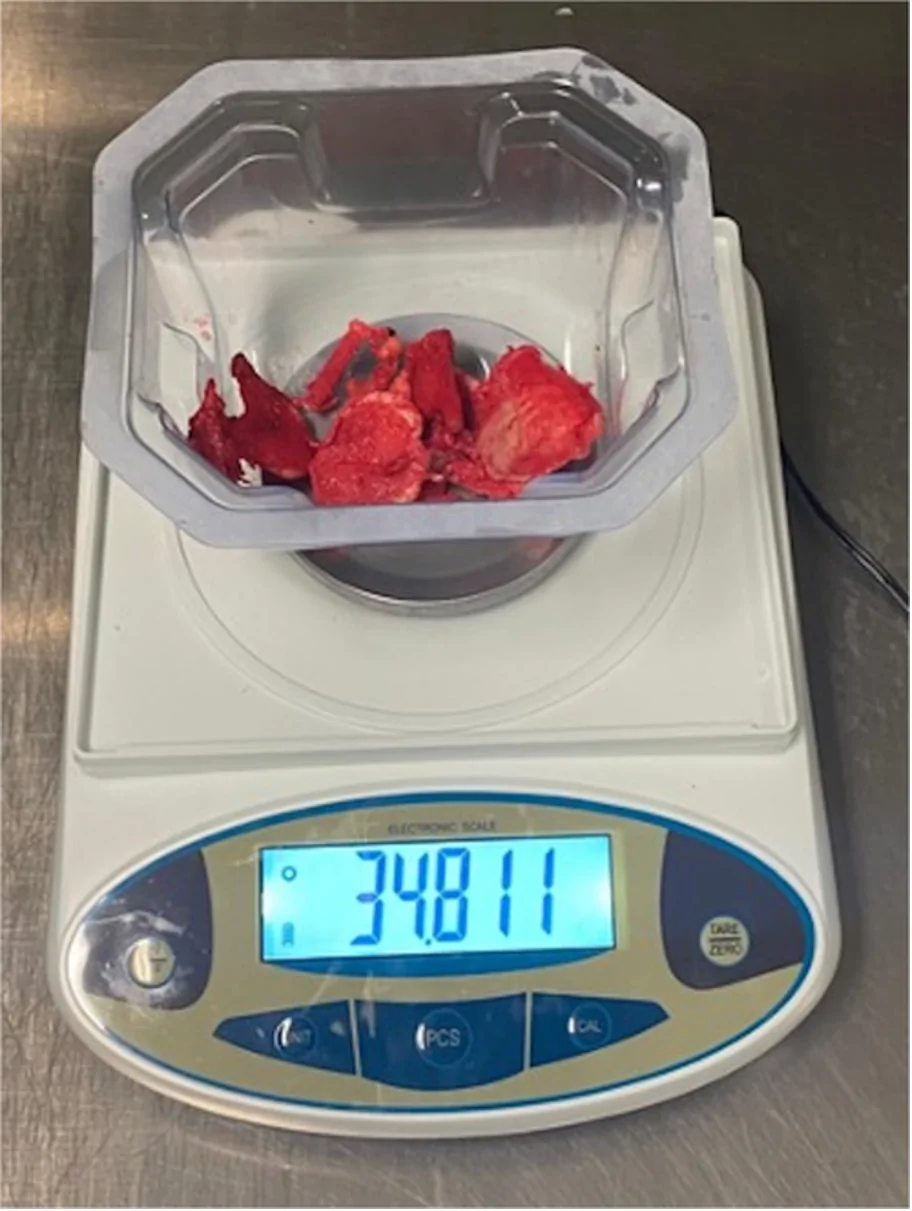
The precision balance used for all the measurements.

The resected synovial tissue and femoral osteophytes were carefully collected using the same standardized technique. All osteophytes from the femur were collected in a single container. Bone from the femoral resections was collected in the same manner. Similarly, Excess cement extruded from the femoral component and the residual cement remaining in the cement gun were also collected. At the end of the procedure, the removed bone (cuts and osteophytes), the removed soft tissues and the remaining cement was weighed using a precision electronic balance with a resolution of 0.0001, after subtracting the weight of the collection container (Figure 2).

The weight of each femoral component size was measured separately using the same precision balance and recorded in a dedicated spreadsheet (Excel®; Microsoft Corp.).

The weight of the removed bone ($W_{\mathrm{bone}\mathrm{cuts}}$) was the sum of the removed bone from the femoral cuts and the osteophytes (and soft tissue if removed). The weight of the implanted cement (IC) was calculated by subtracting the combined weight of the unused cement remaining in the gun and the cement removed from the patient from the total weight of the new cement gun.

As a result, the overall implant weight ($W_{\mathrm{implant}}$) was calculated as follows:

$$W_{\mathrm{implant}}=W_{\mathrm{component}}+0.5\times \mathrm{IC}.$$

A total of 50% of the implanted cement mass was attributed to the femoral component. This arbitrary assumption was necessary to simplify the model. However, previous studies have reported that approximately 10–12 g of cement are typically applied to the femoral component out of a total cement mass of approximately 20–23 g [3, 24]. Moreover, the results of a prospective study on 133 primary TKA indicate that using one 40‐g cement pack for small, medium and large size femoral components was 19.9, 20.8 and 26.0 g, respectively; therefore, 50% of the cement is usually applied to the femoral component, and the range may change according to the component size [26].

### Biomechanical model

**Figure 3:**
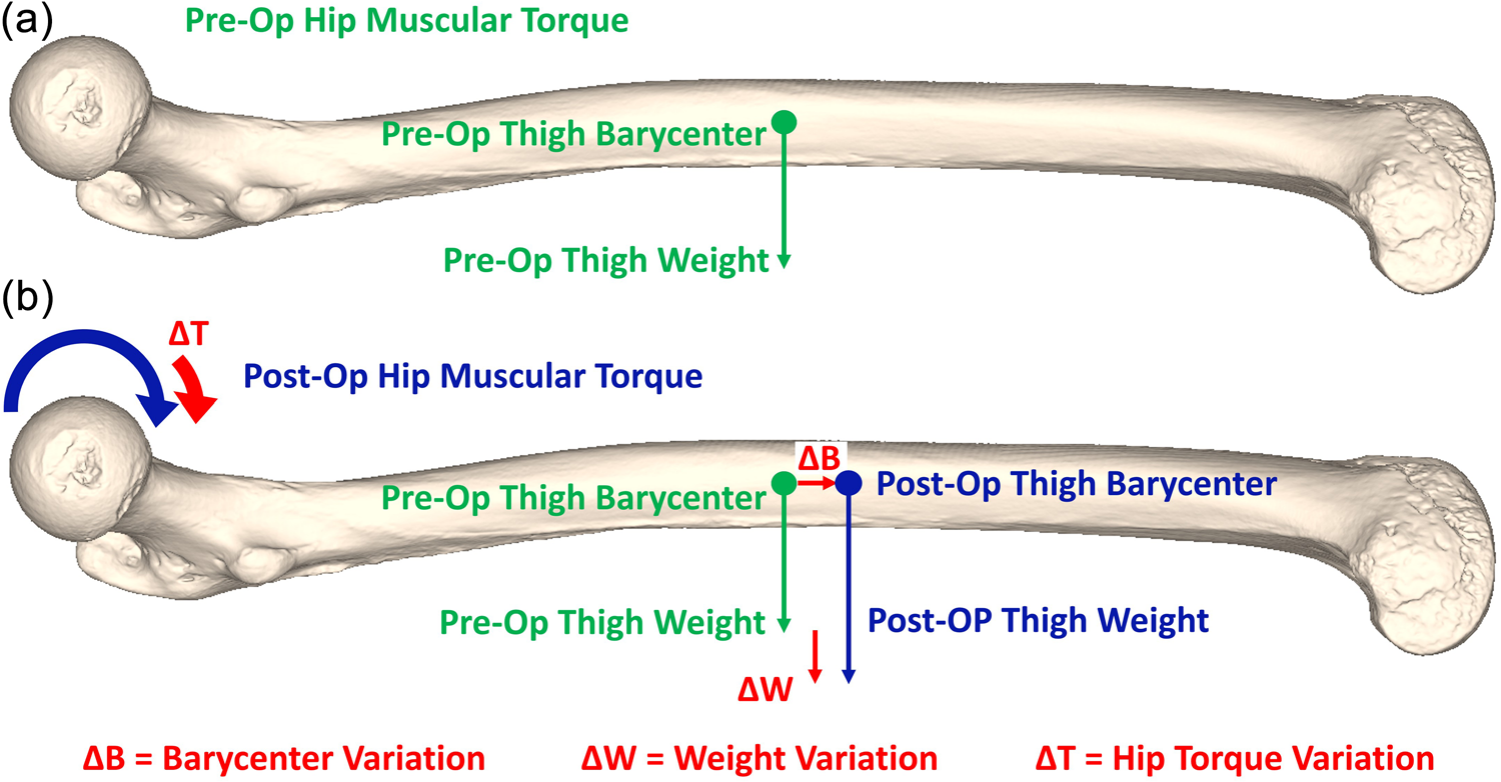
Graphical representation of the biomechanical model used in the current study (soft tissues are not shown for clarity). (a) Pre‐operative model. (b) Post‐operative model. The analysed biomechanical parameters (thigh centre of mass, thigh weight and hip muscular torque) are shown in green for the pre‐operative conditions and in blue for the post‐operative conditions. The changes in these parameters (named, respectively, Δ*B*, Δ*W*, Δ*T*) are reported in red in the figure.

For each patient, a patient‐specific two‐dimensional biomechanical model was developed to quantify the mechanical implications of the increased weight resulting from the replacement of the distal femoral condyles with a cemented metallic implant. As the model development was restricted to the thigh segment, only the femoral component of the TKA was considered. The analytical model was developed to simulate a 90° hip flexion condition (Figure 3). This configuration was selected because hip flexion angles around 90° are associated with the highest lever arm from the hip joint centre to the thigh centre of mass [9]. Functionally, this corresponds to critical daily activities such as sit‐to‐stand and stair ascent, where quadriceps and hip extensors are most challenged. The analysis was performed under quasi‐static conditions and focused on the pre‐operative vs post‐operative thigh centre of mass, weight and hip joint muscular torque [9, 20]. The proposed model represents a first‐order analytical approximation aimed at isolating the effect of implant‐related mass redistribution. It does not incorporate patient‐specific muscle activation, soft tissue characteristics, dynamic gait patterns or three‐dimensional femoral morphology; therefore, the estimated changes should not be interpreted as direct in vivo alterations in hip joint loading.

The pre‐operative configuration of the thigh was modelled as a rigid, homogeneous segment from the most proximal point of the femoral head to the most distal point of the femoral condylesof the femoral condyles. The segment was considered projected onto the sagittal plane, since the primary mechanical effect of the femoral component weight occurs along the longitudinal axis of the femur. Thus, rotational or coronal malalignments contribute minimally to segmental weight distribution, and they were therefore excluded to highlight the mechanical contribution of the implant weight. Coronal and rotational parameters were not included because the current model was specifically designed to isolate the effect of implant‐related mass redistribution. Anthropometric parameters, including thigh segment weight and centre of mass location, were estimated using widely established regression data derived from cadaveric and in vivo analyses [28]. In detail, for each patient, the thigh weight was obtained as a 10% of total body weight, assuming a uniform density distribution prior to implant insertion. The pre‐operative native thigh centre of mass position was defined along the longitudinal axis of the femur, estimated as a fixed percentage of thigh segment length (43.3%) calculated from the proximal end; this location reflects the natural distribution of soft tissues and bone along the thigh segment. The pre‐operative configuration served as the baseline for quantifying the post‐operative biomechanical alterations induced by the implantation of the metallic component. Due to the two‐dimensional formulation of the biomechanical model, all anatomical and morphometric parameters, such as limb deformity, femoral anteversion and bowing, and so on, were assumed negligible for this formulation. The post‐operative configuration consists of the removal of the bony material due to the five standard femoral bone cuts (whose weight was measured intraoperatively), and the inclusion of the additional weight introduced by the metallic femoral component and the corresponding bone cement used during the femoral procedure (also measured intraoperatively). The removed bone and implanted component were assumed to have the same distal thickness, ensuring that post‐operative weight changes arise only from differences in material composition (metal vs. bone), rather than from thickness alterations. Consequently, any resulting differences in the femoral length were considered negligible, and therefore the implant geometry does not alter the length of the thigh segment, which is consistent with clinical reality in TKA. The implant sagittal centre of mass, expressed as distance from the most distal end (femoral condyles), was measured from three‐dimensional implant geometries by computing the centroid of the solid model in the sagittal plane, assuming homogeneous density distribution of the metallic alloy.

### Thigh weight percentage variation (∆*W*_%_)

The variation in thigh segment weight resulting from implantation of the femoral component was quantified by comparing the post‐operative and pre‐operative weights of the thigh. To express the effect of the weight change, the variation was calculated as a percentage change relative to the pre‐operative thigh weight:

$$∆W_{\%}=\frac{W_{\mathrm{implant}}-W_{\mathrm{bone}\;\mathrm{cuts}}}{W_{\mathrm{thigh},\mathrm{pre}‐\mathrm{op}}}\times 100,$$

where $∆W_{\%}\;$is the percentage change of thigh weight, $W_{\mathrm{implant}}$ is the overall weight of the implant, $W_{\mathrm{bone}\mathrm{cuts}}$ is the weight of the bone cuts and $W_{\mathrm{thigh},\mathrm{pre}-\mathrm{op}}$ is the pre‐operative weight of the thigh.

This formulation highlights the proportional impact of the implant weight on the overall thigh segment, independently of patient anatomy and body size. Thus, the resulting percentage variation reflects the relative change of the thigh weight due solely to the prosthetic component. This parameter was subsequently used to quantify the observed changes in centre of mass position and hip joint torque.

### Thigh centre of mass variation (∆B)

**Figure 4:**
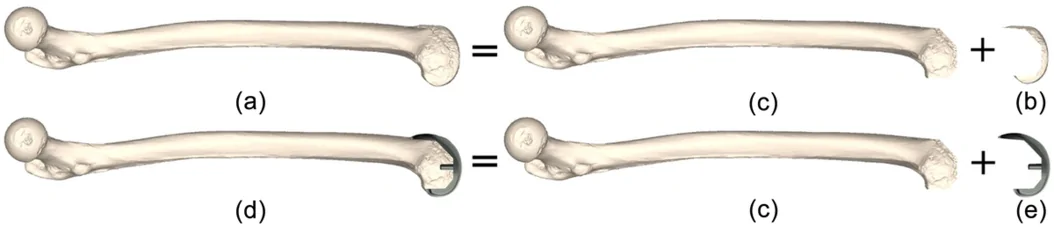
Schematic representation of the approach for the calculation of the centre of mass displacement. (a) Pre‐operative full model. (b) Distal bone cuts. (c) Remaining proximal femoral bone. (d) Post‐operative full model. (e) Simulation of femoral implant and cement.

To compute the position of the thigh centre of mass in both the pre‐operative and post‐operative configurations, a weight‐equivalence principle was applied (Figure 4). In the pre‐operative condition, the thigh segment was first modelled as a single body with its known weight and centre of mass (Figure 4a). The same configuration is also equal to the combination of two sub‐models: the distal bone cuts system (Figure 4b), modelled with its specific weight and centre of mass, and the remaining proximal femoral bone system (Figure 4c).

In the post‐operative condition, the thigh segment was first modelled as a single body with its unknown weight and centre of mass (Figure 4d). Similarly, this configuration is also equal to the combination of two sub‐models: the femoral implant and cement (Figure 4e), and the remaining proximal femoral bone (that is equal to the one in the pre‐operative model, Figure 4c).

Therefore, the final analytical expression that directly provides the overall centre of mass shift of the thigh from from the pre‐operative to the post‐operative condition was derived as

$$∆B=\frac{\left(L_{\mathrm{femur}}-B_{\mathrm{implant}}-B_{\mathrm{thigh},\mathrm{pre}-\mathrm{op}}\right)\;∙\;\left(W_{\mathrm{implant}}-W_{\;\mathrm{bone}\;\mathrm{cuts}}\right)}{W_{\mathrm{thigh},\mathrm{pre}-\mathrm{op}}+W_{\mathrm{implant}}-W_{\;\mathrm{bone}\;\mathrm{cuts}}},$$

where ∆B is the centre of mass shift, $L_{\mathrm{femur}}$ is the length of the femur, $B_{\mathrm{implant}}$ is the centre of mass of the implant, $B_{\mathrm{thigh},\mathrm{pre}-\mathrm{op}}$ is the pre‐operative centre of mass of the thigh, $W_{\mathrm{implant}}$ is the weight of the implant, $W_{\mathrm{bone}\mathrm{cuts}}$ is the weight of the bone cuts and $W_{\mathrm{thigh},\mathrm{pre}-\mathrm{op}}$ is the pre‐operative weight of the thigh.

### Hip flexion muscular torque percentage variation (∆*T*_%_)

The biomechanical effect on the hip joint of the metallic implant insertion was quantified by evaluating the change in hip flexion muscular torque between the pre‐operative and post‐operative configurations induced by the change in thigh weight and barycentric location. In both conditions, the muscular torque was evaluated as the product of the respective thigh segment weight and its lever arm, evaluated as the distance of the centre of mass from the hip joint [9, 20].

To normalize the effect across different patients' anatomies and body size, the variation in hip muscular torque was expressed as a percentage change relative to the pre‐operative condition using the following equation:

$$∆T_{\%}=\frac{W_{\mathrm{post}-\mathrm{op}}\times B_{\mathrm{post}-\mathrm{op}}-W_{\;\mathrm{pre}-\mathrm{op}}\times B_{\mathrm{pre}-\mathrm{op}}}{W_{\;\mathrm{pre}-\mathrm{op}}\times B_{\mathrm{pre}-\mathrm{op}}}\times 100,$$

where $∆T_{\%}\;$is the percentage change of hip flexion muscular torque, $W_{\mathrm{post}-\mathrm{op}}$ is the overall post‐operative thigh weight, $B_{\mathrm{post}-\mathrm{op}}$ is the overall post‐operative thigh centre of mass, $W_{\mathrm{pre}-\mathrm{op}}$ is the overall pre‐operative thigh weight and $B_{\mathrm{pre}-\mathrm{op}}$ is the overall pre‐operative thigh centre of mass.

### Statistical analysis

To evaluate the variability in the biomechanical parameters, a statistical analysis was conducted, investigating their distribution and dispersion among the different patients. Pre‐operative and post‐operative values were compared using a paired Student's *t* test, with statistical significance set at a two‐tailed *p* value < 0.05. Scatter plots were generated for the primary outcomes, including percentage variation in thigh weight, centre of mass shift and percentage change in hip muscular torque, to visually assess data distribution. Mean values, SDs and ranges, including 95% confidence intervals where appropriate, were computed for each parameter. To further investigate distribution characteristics, each of the three primary biomechanical outcomes was stratified into predefined ranges. The number of patients falling within each range was quantified for all three parameters. This categorical analysis provided additional information on whether the distributions tended to cluster homogeneously or asymmetrically and allowed identification of potential patterns or subgroups within the cohort. A linear regression analysis was also performed to evaluate the relationship between patient BMI and the percentage increase in hip muscular torque.

Statistical analyses were performed using MATLAB R2024a (The MathWorks Inc.).

## RESULTS

### Population

A post hoc power analysis was performed considering an alpha level of 0.05 and a sample size of 70 patients. The effect sizes were calculated from the observed mean changes and corresponding SDs for the main biomechanical outcomes, including thigh weight variation, centre of mass displacement, and estimated hip muscular moment variation. The analysis demonstrated that the study population provided adequate statistical power (>80%) to detect the observed changes in all investigated parameters. Statistical power was calculated using G*Power (Version 3.1.9.6).

The mean pre‐operative HKA was 173.8° (SD 5.5). The mean femoral length (FML) was 436.6 mm (SD 33.4), the mean intra‐medullary canal diameter was 29.2 mm (SD 2.9), and the mean thigh diameter was 163 mm (SD 18.7). Considering the femoral measurements, the mean difference between the implanted femoral construct and the resected femoral bone was 199.1 g (SD 40.1, Table 1).

**Table 1:** Summary of the average values for femoral bone cuts (including osteophytes), femoral component implanted (including cement) and the differences between those values.

Femoral bone cuts (SD)	Femoral component implanted (SD)	∆ femoral weight (SD)
40.5 g (12.0)	239.6 g (44.1)	199.1 (40.1)

Abbreviation: SD, standard deviation.

**Figure 5:**
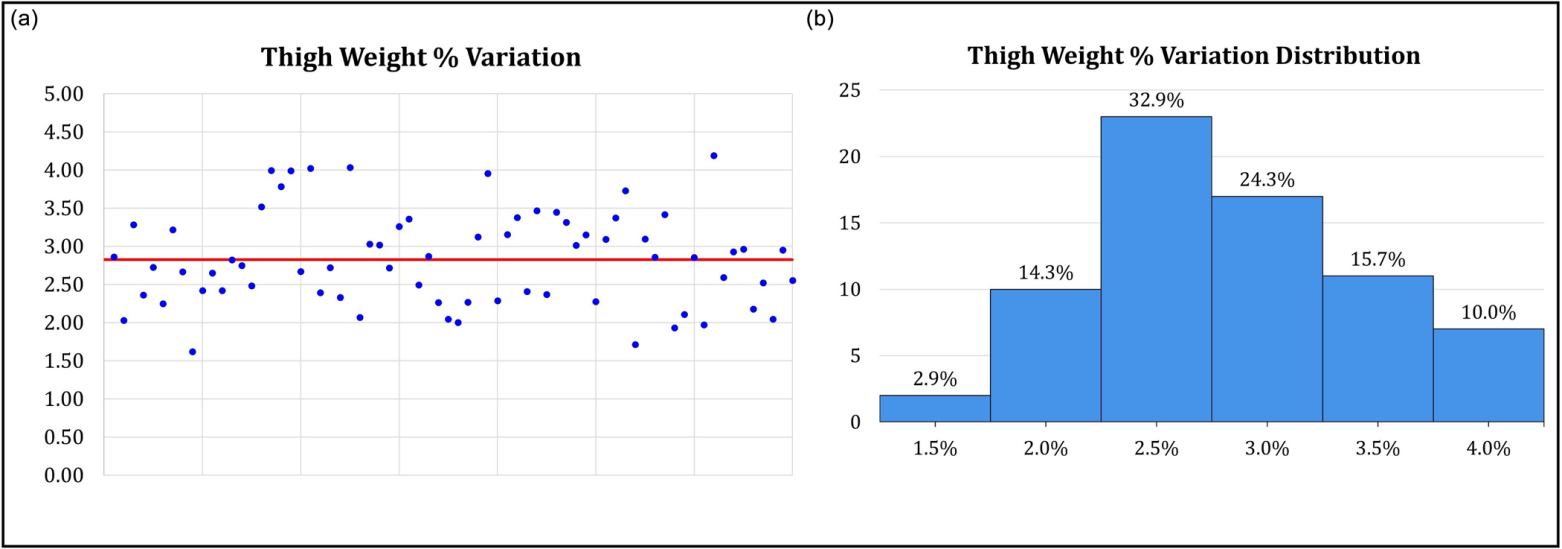
Thigh weight percentage variation (a) and distribution of variation (b).

The values of $∆W_{\%}$ are reported in Figure 5. Femoral component implantation significantly increased thigh weight in all patients; the insertion of a prosthesis consistently increased thigh weight, with an average increase of approximately 3% (mean 2.8%, SD 0.6%, and range: 1.6%–4.2%, *p* < 0.05).

A categorical distribution analysis was performed for Δ*W*_%_, grouping results into defined percentage bands to characterize whether weight increases tended to cluster within specific ranges (Figure 5b). The bins were spaced at 0.5% intervals, with a ±0.25% margin around each value. The distribution showed that thigh weight increased by approximately 2.5% in almost one‐third of patients.

### Thigh centre of mass variation (∆B)

**Figure 6:**
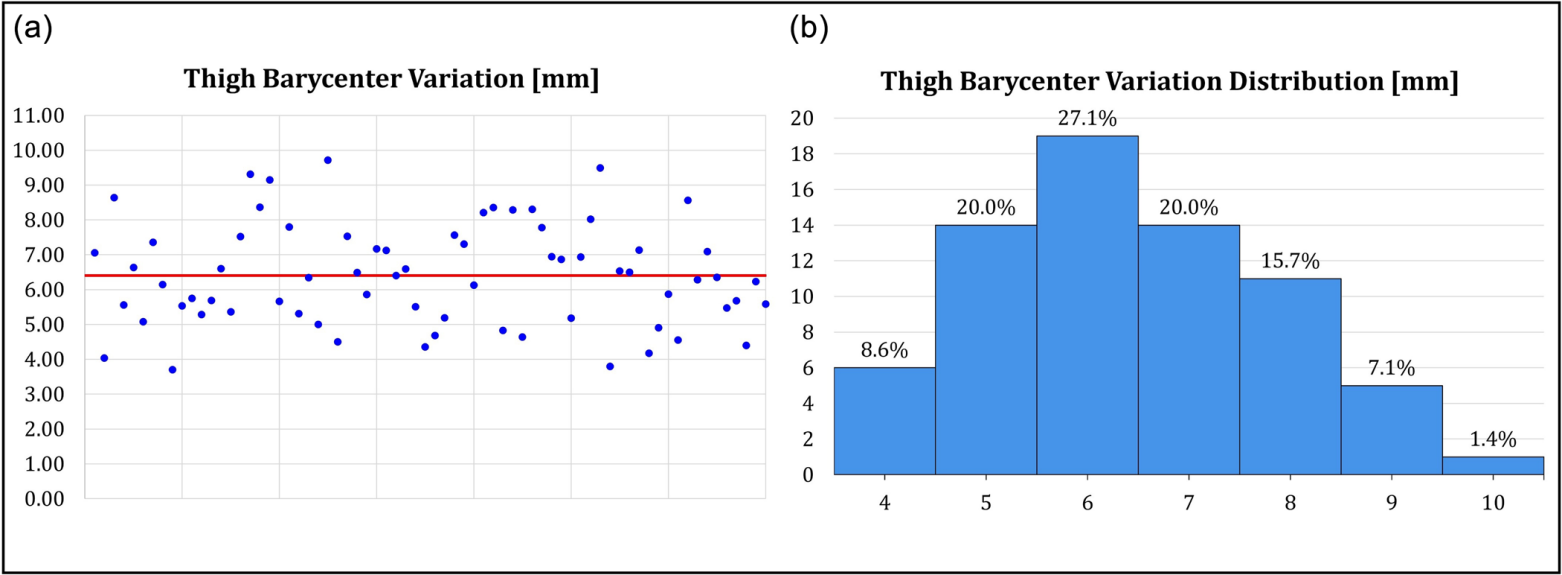
Thigh centre of mass variation (a) and distribution of variation (b).

The values of ∆B are reported in Figure 6. Femoral component implantation induced a significant distal shift of the thigh centre of mass in all patients, with a mean displacement of 6.4 mm (SD 1.5 mm; range: 3.7–9.7 mm, *p* < 0.05). The distribution of ∆B values was also analysed through discrete categories, and the number of patients in each category was quantified. The categories were spaced at 1‐mm intervals, with a ±0.5‐mm margin around each value (Figure 6b). The distribution showed that 70% of patients had a distal centre of mass shift between 5.5 and 9.5 mm, whereas the most frequent category was centred around 6 mm (27.1%).

### Hip flexion muscular torque percentage variation (∆*T*_%_)

**Figure 7:**
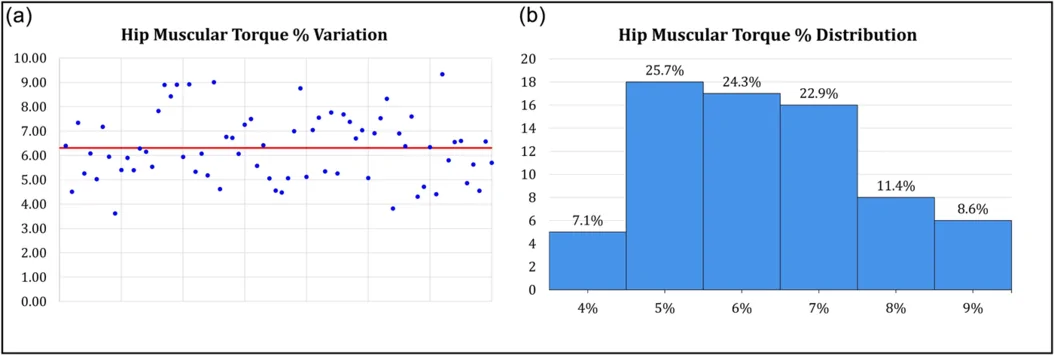
Hip muscle moment percentage (%) variation (a) and distribution of variation (b).

The values of $∆T_{\%}$ are reported in Figure 7. It can be observed that, in every patient, the insertion of a prosthesis consistently induces a significant increase in hip muscular torque, with an average increase of approximately 6% (mean 6.3%, SD 1.4%, and range: 3.6%–9.3%, *p* < 0.05).

**Figure 8:**
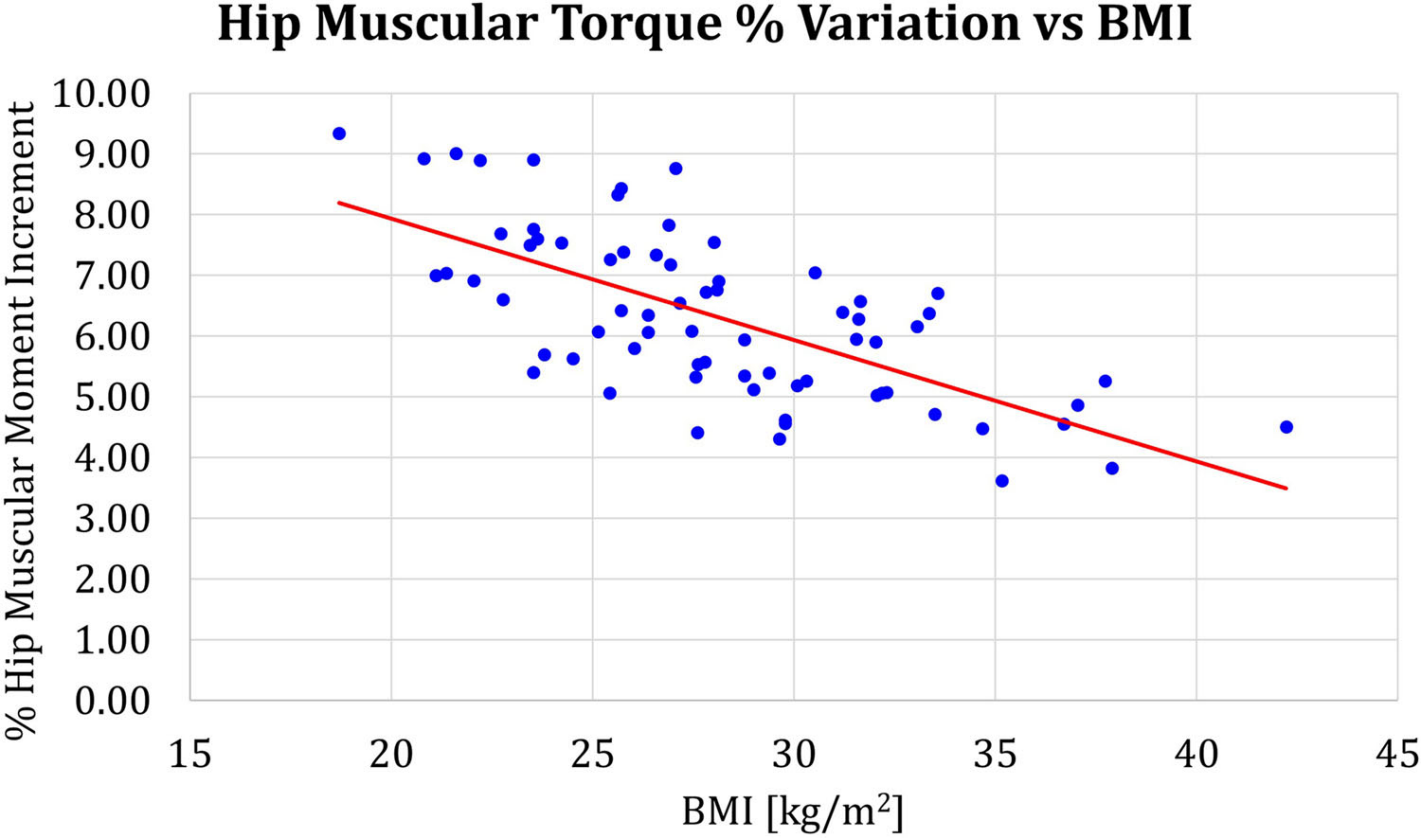
Hip muscle moment variation (%) and its correlation with body mass index (BMI).

$∆T_{\%}$ values were also grouped into categorical bins to assess how increases were distributed across the patient population. The categories were spaced at 1% intervals, with a ±0.5% margin around each value (Figure 7b). The resulting categories highlighted that approximately 20% of patients had an increase of 7.5% or greater. A scatterplot of $∆T_{\%}$ versus BMI was generated to assess the relationship between BMI and the percentage increase in the estimated hip flexion moment (Figure 8). A linear regression analysis demonstrated that patients with lower BMI exhibited proportionally larger increases in hip flexor torque. The regression slope was −0.2, indicating that each 1‐kg/m^2^ decrease in BMI was associated with a 0.2‐percentage‐point greater increase in $∆T_{\%}$.

All results are summarized in Table 2.

**Table 2:** Overall results of the biomechanical model in terms of mean, standard deviation (SD) range and 95% confidence interval.

Parameter	Mean	SD	Range		95% confidence interval	
			Min	Max	Min	Max
Δ*W*%	2.8%	0.6%	1.6%	4.2%	2.7%	3.0%
Δ*B*	6.4 mm	1.5 mm	3.7 mm	9.7 mm	6.0 mm	6.7 mm
Δ*T*%	6.3%	1.4%	3.6%	9.3%	6.0%	6.6%

## DISCUSSION

The most important finding of the study was an increase in femoral weight of approximately 200 g after TKA. Considering that standard anatomical references estimate the average femoral weight to range from approximately 270 to 550 g in adults [22, 27], depending on sex and body size, the increase observed after TKA implantation is substantial. Gibon et al. in a previous study described a mean increase in weight after TKA implantation of 266.7 g (SD 35.1) and 279.1 g (SD 48.7), depending on the brand [6]. Similarly, Zhu et al. reported a mean increase in weight of 298.9 g (SD 63.7) [29]. However, both studies did not separate the femoral and tibial components and did not evaluate the possible biomechanical consequences of the increased femoral weight after TKA implantation.

In this study, a biomechanical model of the thigh and hip flexor muscles was developed to estimate the consequences in simple activities (such as ascending stairs) of the increased femoral weight following TKA implantation. The mean increase in thigh weight was 2.8% (SD 0.6), with 72.9% of patients showing an increase between 2.5% and 3.5% (Figure 5). This increase in thigh weight resulted in a shift in the centre of variation of the femoral centre of mass (Figure 3), which was more distal in all the patients, with an average of 6.4 mm (SD 1.5) and 71.3% of patients showing a distal shift greater than 6 mm (Figure 6).

Although the changes in weight and centre of mass may appear minimal, they have a non‐negligible impact on the increase in the muscular moment required by the patient, particularly in individuals with a low BMI. In this biomechanical model, the analysed scenario represents a biomechanical extreme case, which frequently occurs in daily activities. It corresponds to a condition in which the hip is flexed at 90°, such as during rising from a chair or climbing stairs, where the hip flexor muscles are subjected to greater demand due to the increased weight and lever arm. In this situation, the mean increase in hip muscular moment was 6.4%, with more than 90% of patients showing an increase greater than 5%. To provide a practical interpretation of this increase, in an 80‐kg adult the hip muscles during simple activities typically generate moments in the range of approximately 15–25 Nm [9]. A 6.3% increment therefore corresponds to an additional demand of about 1–1.6 Nm. Assuming an average hip flexor moment arm of 4 cm, this corresponds to an increase in muscular force of approximately 25–40 N.

Biomechanical analyses have shown that hip joint reaction forces can exceed six times body weight, reflecting the significant muscular effort required to stabilize and elevate the trunk and lower limb during simple activities, such as raising a chair [14]. Furthermore, greater hip flexion angles during seat‐off further increase joint contact forces and the corresponding muscular demand, due to the lengthened lever arm and altered moment distribution across the hip [10]. In this context, the hip flexor muscles—particularly the iliopsoas and rectus femoris—play a key role—particularly the iliopsoas and rectus femoris—play a key role in controlling limb trajectory and generating the required moments, with their activation increasing substantially as segmental mass or inertia increases.

This study has several limitations that should be considered when interpreting the results. First, it is based on a two‐dimensional biomechanical model of the thigh, which is quite accurate but did not consider differences or deformities in the coronal or sagittal planes, which may lead to small variations in the results. The thigh was modelled as a rigid unit, but there is a wide variation across the population. However, the biomechanical model was constructed based on data from the literature [9]. A strong association between $∆T_{\%}$ on BMI was observed, which can be biomechanically explained by the disproportion between patient anthropometric variability and implant variability. Furthermore, the hip muscles were considered as a single unit, with no evaluation of the role of the individual muscles. However, the biomechanical model was intentionally kept simple by reducing the number of variables; therefore, limb deformity, femoral anteversion, and femoral bowing were not considered. Only one implant system was evaluated; therefore, the present findings should not be generalized to all TKA designs, as implant‐specific differences in geometry, material composition and mass distribution may influence the magnitude of the observed biomechanical effects. Moreover, PS and MC implants were analysed together despite small differences in weight. The analytical equations used in this study have not been validated against experimental or dynamic biomechanical data. Therefore, the estimated increase in hip muscular moment should be interpreted as a theoretical consequence of implant‐related mass redistribution rather than a direct quantification of in vivo hip loading.

Lastly, the direct impact of the increase in thigh weight on the patellofemoral joint reaction forces was not calculated, due to the complexity of the model (introduction of tibial baseplate, component rotation, other muscular structures, etc.). However, considering the potential implications in the patellofemoral joint, this aspect will be investigated as the next step of the study.

## CONCLUSION

This preliminary biomechanical study suggests that TKA implantation may increase thigh weight and induce a distal shift of the thigh centre of mass, resulting in a measurable increase in hip muscular moment. Although these changes were modest, they may be biomechanically relevant during functional activities, and further modelling and clinical studies are needed to clarify their potential impact on joint loading and patient outcomes.

## AUTHOR CONTRIBUTIONS

**Federica Rosso**: Conceptualization; writing; review and editing. **Mattia Sisella**: Methods; validation. **Francesco Romano**: Investigation; validation. **Tommaso Nosenzo**: Investigation; validation. **Roberto Rossi**: Conceptualization; supervision; review and editing. **Bernardo Innocenti**: Conceptualization; supervision; review and editing.

## FUNDING INFORMATION

The authors have no funding to report.

## CONFLICT OF INTEREST STATEMENT

Federica Rosso reports receiving research grants from Medacta and serving as a committee member of SIAGASCOT (Italian Society of Arthroscopy, Knee, Upper Limb, Sports, Cartilage and Orthopaedic Technologies) and an active member of the European Knee Society. Roberto Rossi reports receiving payment from Arthrex, Inc. for serving as a presenter or speaker; receiving intellectual property royalties from Lima Enovis and payment for serving as a presenter or speaker; and receiving payment from Zimmer for serving as a consultant and presenter or speaker. He also serves as a committee member of SIAGASCOT, a member of the European Knee Society, an international member of the American Association of Hip and Knee Surgeons and Deputy Editor of *JAAOS Global Research & Reviews*. Bernardo Innocenti serves as a council member of the European Society of Biomechanics, a member of the European Knee Society, and an editorial board member of the *Journal of Arthroplasty* and *Knee Surgery, Sports Traumatology, Arthroscopy*. He also reports receiving payment from Link Orthopaedic for serving as a presenter, speaker and consultant, and from Adler Ortho for serving as a presenter, speaker and consultant. The remaining authors declare no conflicts of interest.

## ETHICS STATEMENT

The study was conducted in alignment with the ethical principles outlined in the 1964 Declaration of Helsinki as well as the Health Insurance Portability and Accountability Act (HIPAA) guidelines. The Institutional Review Board (IRB) at the author's institution classified this research as exempt from IRB oversight (biomechanical model on anonymized patients' data).

## Data Availability

Data are available on request from the authors.

## References

[1] Adouni M , Alkhatib F , Hajji R , Faisal TR . Effects of overweight and obesity on lower limb walking characteristics from joint kinematics to muscle activations. Gait Posture. 2024;113:337–344. 10.1016/j.gaitpost.2024.06.024 39032386

[2] Beswick AD , Wylde V , Gooberman‐Hill R . Interventions for the prediction and management of chronic postsurgical pain after total knee replacement: systematic review of randomised controlled trials. BMJ Open. 2015;5(5):e007387. 10.1136/bmjopen-2014-007387 PMC4431062 25967998

[3] Bösebeck H , Holl A‐M , Ochsner P , Groth M , Stippich K , Nowakowski AM , et al. Cementing technique for total knee arthroplasty in cadavers using a pastry bone cement. J Orthop Surg Res. 2021;16(1):417. 10.1186/s13018-021-02436-z PMC8247244 34210335

[4] Browning RC , Modica JR , Kram R , Goswami A . The effects of adding mass to the legs on the energetics and biomechanics of walking. Med Sci Sports Exerc. 2007;39(3):515–525. 10.1249/mss.0b013e31802b3562 17473778

[5] Cottino U , Rosso F , Pastrone A , Dettoni F , Rossi R , Bruzzone M . Painful knee arthroplasty: current practice. Curr Rev Musculoskelet Med. 2015;8(4):398–406. 10.1007/s12178-015-9296-5 PMC4630224 26400422

[6] Gibon E , Mouton A , Passeron D , Le Strat V , Graff W , Marmor S . Doctor, what does my knee arthroplasty weigh? J Arthroplasty. 2014;29(11):2091–2094. 10.1016/j.arth.2014.07.012 25113782

[7] Giovanoulis V , Vasiliadis AV , Andriollo L , Gregori P , Dretakis K , Koutserimpas C , et al. Functional alignment in robotic total knee arthroplasty provides favourable outcomes and minimal early revisions: a systematic review and meta‐analysis. Knee Surg Sports Traumatol Arthrosc. 2026;34:3362–3376. 10.1002/ksa.70226 PMC13502434 41358691

[8] Gunaratne R , Pratt DN , Banda J , Fick DP , Khan RJK , Robertson BW . Patient dissatisfaction following total knee arthroplasty: a systematic review of the literature. J Arthroplasty. 2017;32(12):3854–3860. 10.1016/j.arth.2017.07.021 28844632

[9] Hart HF , Patterson BE , Crossley KM , Culvenor AG , Khan MCM , King MG , et al. May the force be with you: understanding how patellofemoral joint reaction force compares across different activities and physical interventions‐a systematic review and meta‐analysis. Br J Sports Med. 2022;56(9):521–530. 10.1136/bjsports-2021-104686 35115309

[10] Inai T , Takabayashi T , Edama M , Kubo M . Effect of hip joint angle at seat‐off on hip joint contact force during sit‐to‐stand movement: a computer simulation study. Biomed Eng Online. 2018;17(1):177. 10.1186/s12938-018-0610-5 PMC6267796 30497482

[11] Kakiage H , Hatayama K , Nonaka S , Terauchi M , Saito K , Takase R , et al. Stair climbing ability and postoperative activity in patient‐reported outcomes after CR‐TKA are more related to handgrip strength than sagittal knee stability. Arch Orthop Trauma Surg. 2025;145(1):113. 10.1007/s00402-024-05678-8 39776240

[12] Kim HK , Lu S‐H , Lu T‐W , Chou L‐S . Contribution of lower extremity muscles to center of mass acceleration during walking: Effect of body weight. J Biomech. 2023;146:111398. 10.1016/j.jbiomech.2022.111398 36459848

[13] Klug A , Gramlich Y , Rudert M , Drees P , Hoffmann R , Weißenberger M , et al. The projected volume of primary and revision total knee arthroplasty will place an immense burden on future health care systems over the next 30 years. Knee Surg Sports Traumatol Arthrosc. 2021;29(10):3287–3298. 10.1007/s00167-020-06154-7 PMC7362328 32671435

[14] Layton R , Messenger N , Stewart T . Characteristics of hip joint reaction forces during a range of activities. Med Eng Phys. 2022;108:103894. 10.1016/j.medengphy.2022.103894 36195363

[15] Lewis PL , Graves SE , de Steiger RN , Campbell DG , Peng Y , Hatton A , et al. Does knee prosthesis survivorship improve when implant designs change? Findings from the Australian Orthopaedic Association National Joint Replacement Registry. Clin Orthop Relat Res. 2020;478(6):1156–1172. 10.1097/CORR.0000000000001229 PMC7319368 32324669

[16] Mathis DT , Hauser A , Iordache E , Amsler F , Hirschmann MT . Typical pain patterns in unhappy patients after total knee arthroplasty. J Arthroplasty. 2021;36(6):1947–1957. 10.1016/j.arth.2021.01.040 33583666

[17] Mathis DT , Tschudi S , Amsler F , Hauser A , Rasch H , Hirschmann MT . Correlations of typical pain patterns with SPECT/CT findings in unhappy patients after total knee arthroplasty. Knee Surg Sports Traumatol Arthrosc. 2022;30(9):3007–3023. 10.1007/s00167-021-06567-y PMC9418274 33864469

[18] Miura K , Ohkoshi Y , Ino T , Ukishiro K , Kawakami K , Suzuki S , et al. Kinematics and center of axial rotation during walking after medial pivot type total knee arthroplasty. J Exp Orthop. 2020;7(1):72. 10.1186/s40634-020-00286-y PMC7522154 32986185

[19] Nam CH , Lee SC , Kim JH , Ahn HS , Baek JH . Robot‐assisted total knee arthroplasty improves mechanical alignment and accuracy of component positioning compared to the conventional technique. J Exp Orthop. 2022;9(1):108. 10.1186/s40634-022-00546-z PMC9613830 36302997

[20] Özkaya N , Leger D , Goldsheyder D , Nordin M . Fundamentals of biomechanics: equilibrium, motion, and deformation. Cham: Springer International Publishing; 2017.

[21] Planckaert C , Larose G , Ranger P , Lacelle M , Fuentes A , Hagemeister N . Total knee arthroplasty with unexplained pain: new insights from kinematics. Arch Orthop Trauma Surg. 2018;138(4):553–561. 10.1007/s00402-018-2873-5 29322318

[22] Pomeroy E , Mushrif‐Tripathy V , Kulkarni B , Kinra S , Stock JT , Cole TJ , et al. Estimating body mass and composition from proximal femur dimensions using dual energy x‐ray absorptiometry. Archaeol Anthropol Sci. 2019;11(5):2167–2179. 10.1007/s12520-018-0665-z PMC6743672 31565085

[23] Powers CM , Ho K‐Y , Chen Y‐J , Souza RB , Farrokhi S . Patellofemoral joint stress during weight‐bearing and non‐weight‐bearing quadriceps exercises. J Orthop Sports Phys Ther. 2014;44(5):320–327. 10.2519/jospt.2014.4936 24673446

[24] Rodríguez‐Collell JR , Mifsut D , Ruiz‐Sauri A , Rodríguez‐Pino L , González‐Soler EM , Valverde‐Navarro AA . Improving the cementation of the tibial component in knee arthroplasty: a study of four techniques in the cadaver. Bone Joint Res. 2021;10(8):467–473. 10.1302/2046-3758.108.BJR-2020-0524.R1 PMC8414436 34340533

[25] Sacco R , Tecame A , Lalevée M , Perrier A , Massa E , Kouyoumdjian P , et al. Robotic vs. conventional total knee arthroplasty over two decades: evolving trends toward personalised alignment without significant clinical superiority in predominantly mild varus deformity—a systematic review of RCTs. J Exp Orthop. 2025;12(4):e70452. 10.1002/jeo2.70452 PMC12670295 41341124

[26] Satish BRJ , Thadi M , Thirumalaisamy S , Sunil A , Basanagoudar PL , Leo B . How much bone cement is utilized for component fixation in primary cemented total knee arthroplasty? Arch Bone Jt Surg. 2018;6(5):381–389. PMC6168233 30320178

[27] Singh S , Singh SP . Weight of the femur—a useful measurement for identification of sex. Cells Tissues Organs. 1974;87(1):141–145. 10.1159/000144166 4855411

[28] Winter DA . Biomechanics and motor control of human movement. Hoboken, NJ: Wiley; 2009.

[29] Zhu Z , Tang T , Pan S , Sun Z , Huang C , Ruan R , et al. Effect of knee joint weight change on knee function recovery and gait after total knee arthroplasty. BMC Musculoskelet Disord. 2022;23(1):694. 10.1186/s12891-022-05647-5 PMC9306069 35869453

